# The prognostic value of serum C-reactive protein-bound serum amyloid A in early-stage lung cancer

**DOI:** 10.1186/s40880-015-0039-1

**Published:** 2015-08-10

**Authors:** Xue-Yan Zhang, Ge Zhang, Ying Jiang, Dan Liu, Man-Zhi Li, Qian Zhong, Shan-Qi Zeng, Wan-Li Liu, Mu-Sheng Zeng

**Affiliations:** State Key Laboratory of Oncology in South China, Collaborative Innovation Center for Cancer Medicine, Sun Yat-sen University Cancer Center, Guangzhou, Guangdong 510060 P.R. China; Department of Experimental Research, Sun Yat-sen University Cancer Center, Guangzhou, Guangdong 510060 P.R. China; Department of Pathogenic Biology, Guangzhou Hoffmann Institute of Immunology, School of Basic Sciences, Guangzhou Medical University, Guangzhou, Guangdong 510182 P.R. China; Department of Microbial and Biochemical Pharmacy, School of Pharmaceutical Sciences, Sun Yat-sen University, Guangzhou, Guangdong 510006 P.R. China; State Key Laboratory of Proteomics, Beijing Proteome Research Center, Beijing Institute of Radiation Medicine, Beijing, 102206 P.R. China; Department of Clinical Laboratory, Sun Yat-sen University Cancer Center, Guangzhou, Guangdong 510060 P.R. China; Department of Gastrointestinal Surgery, Guangzhou Digestive Disease Center, Guangzhou First People’s Hospital, Guangzhou Medical University, Guangzhou, Guangdong 510180 P.R. China

**Keywords:** Serum C-reactive protein-bound serum amyloid A, Lung cancer, Prognostic marker, Proteomic analysis

## Abstract

**Background:**

Elevated levels of serum C-reactive protein (CRP) have been reported to have prognostic significance in lung cancer patients. This study aimed to further identify CRP-bound components as prognostic markers for lung cancer and validate their prognostic value.

**Methods:**

CRP-bound components obtained from the serum samples from lung cancer patients or healthy controls were analyzed by differential proteomics analysis. CRP-bound serum amyloid A (CRP-SAA) was evaluated by co-immunoprecipitation (IP). Serum samples from two independent cohorts with lung cancer (retrospective cohort, 242 patients; prospective cohort, 222 patients) and healthy controls (159 subjects) were used to evaluate the prognostic value of CRP-SAA by enzyme-linked immunosorbent assay.

**Results:**

CRP-SAA was identified specifically in serum samples from lung cancer patients by proteomic analysis. CRP binding to SAA was confirmed by co-IP in serum samples from lung cancer patients and cell culture media. The level of CRP-SAA was significantly higher in patients than in healthy controls (0.37 ± 0.58 vs. 0.03 ± 0.04, *P* < 0.001). Elevated CRP-SAA levels were significantly associated with severe clinical features of lung cancer. The elevation of CRP-SAA was associated with lower survival rates for both the retrospective (hazard ration [HR] = 2.181, 95% confidence interval [CI] = 1.641–2.897, *P* < 0.001) and the prospective cohorts (HR = 2.744, 95% CI = 1.810–4.161, *P* < 0.001). Multivariate Cox analysis showed that CRP-SAA was an independent prognostic marker for lung cancer. Remarkably, in stages I–II patients, only CRP-SAA, not total SAA or CRP, showed significant association with overall survival in two cohorts. Moreover, univariate and multivariate Cox analyses also showed that only CRP-SAA could be used as an independent prognostic marker for early-stage lung cancer patients.

**Conclusion:**

CRP-SAA could be a better prognostic marker for lung cancer than total SAA or CRP, especially in early-stage patients.

## Background

Lung cancer is the most common cause of cancer-associated death worldwide [1]. In China, the incidence and mortality of lung cancer are the highest among all cancers [2–4]. Early diagnosis and appropriate treatment are key factors for the improvement of prognosis. Predictive and prognostic biomarkers are needed to divide early-stage lung cancer patients into subgroups [5] and can improve medical decision-making in delivering the most suitable treatment.

Recently, chronic inflammation has been found to be associated with tumor progression, and many inflammatory factors could serve as prognostic biomarkers for some tumors [6, 7]. Lung cancer presents as a chronic inflammatory disease [8, 9]. Several studies have suggested that the elevation of serum C-reactive protein (CRP) could be used as a prognostic factor for lung cancer [10–12].

CRP, a classical member of the pentraxin family, has high affinity for many types of autologous and extrinsic ligands. Autologous ligands include damaged cell membranes, apoptotic cells, plasma lipoproteins, phospholipids, ribonucleoprotein particles, extracellular matrix proteins, and Fc-γ receptors. Extrinsic ligands include various constituents of many microorganisms. Once aggregated or bound to macromolecular ligands, CRP activates the complement system to form membrane attack complex, which in turn attacks target molecules or cells. Therefore, CRP plays a key scavenger role in the clearance of abnormal cells or apoptotic cells [13, 14]. CRP is secreted by hepatocytes in response to the inflammatory cytokines produced by the tumor microenvironment [15], and CRP can enter the tumor microenvironment through the circulation, where it binds to a variety of autologous and extrinsic ligands and plays a key role in the clearance of tumor cells [16].

As CRP has the ability to bind to various ligands, a subset of the circulating CRP pool may exist in the form of CRP complexes. Because elevated circulating levels of CRP have been frequently found in lung cancer patients [10–12], we hypothesized that CRP may bind to ligands expressed by lung cancer cells or tumor-associated cells and that CRP-bound components in the serum of lung cancer patients may be different from those of healthy subjects. Therefore, CRP-bound complexes in the serum may be potential prognostic biomarkers of lung cancer.

In an effort to identify possible novel biomarkers to further refine the prognostic prediction accuracy of serum CRP levels for lung cancer, we used differential proteomics technology to identify a specific type of CRP complex in serum samples from lung cancer patients. We also evaluated the value of this CRP complex as a potential prognostic marker in two independent cohorts of lung cancer patients.

## Methods

### Patients and serum samples

Serum samples were collected from two independent cohorts of lung cancer patients recruited from the Sun Yat-sen University Cancer Center (SYSUCC). Patients recruited for the retrospective cohort (*n* = 242) were first diagnosed and treated between 2002 and 2004, and follow-up data from these patients were available up to 2010. Patients recruited for the prospective cohort (*n* = 222) were first diagnosed and treated between 2008 and 2010, and they were followed up to 2014. Detailed patient characteristics are described in Table 1. Serum samples were also obtained from 159 healthy controls recruited from SYSUCC staff volunteers between 2008 and 2010.

**Table 1:** Clinical characteristics of the recruited patients with lung cancer

Characteristic	Retrospective cohort	Prospective cohort
Total (cases)	222	242
Sex [cases (%)]		
Male	169 (76.1)	176 (72.7)
Female	53 (23.9)	66 (27.3)
Age (years)		
Median	59	59
Range	28–91	31–79
Tumor clinical stage^a^ [cases (%)]		
I	37 (16.6)	39 (16.1)
II	43 (19.4)	40 (32.6)
III	71 (32.0)	99 (40.9)
IV	71 (32.0)	64 (26.4)

Patient characteristics were recorded at the time point of blood withdrawal for serum analysis.

^a^Tumor staging was performed according to the seventh edition of the International Lung Cancer Staging published by the Union for International Cancer Control (UICC) and International Association for the Study of Lung Cancer (IASLC).

The selection criteria for lung cancer patients were as follows: histologically confirmed lung cancer, complete documentation of medical history, primary lung cancer features and disease course, completeness of follow-up examinations, and absence of systemic treatment for at least 6 weeks before blood withdrawal. Clinical stage was assessed according to the seventh edition of the Lung Cancer Staging International Division, which was published by the Union for International Cancer Control (UICC) and the International Association for the Study of Lung Cancer (IASLC) in 2009. To use these serum samples for research purposes, prior informed consent from the patients and approval from the Institute Research Ethics Committee of SYSUCC were obtained. The selection criteria for healthy controls were as follows: good physical status, no acute or chronic disease, and not taking any medication.

Serum samples were collected and processed at SYSUCC Biobank according to standard procedures. In brief, venous blood was drawn into serum tubes, clotted at room temperature for 1 h, and subsequently centrifuged at 2,500×*g* for 10 min. Serum was collected, distributed into 100 μL aliquots, and immediately stored at −80°C. Repeated freeze–thaw cycles were avoided for all serum samples.

### Serum proteomic profiling

#### CRP complex purification from mixed serum

Serum samples from 10 lung cancer patients and from 10 healthy controls were mixed, respectively. CRP complexes were purified from the mixed cancer serum or mixed control serum using the following method. In brief, after centrifugation at 10,000×*g* for 10 min at 4°C, 500 μL of serum was diluted in 500 μL of buffer (20 mmol/L Tris–HCl, 150 mmol/L NaCl, 5 mmol/L CaCl_2_, 0.1% Nonidet P-40, pH 7.4), and CRP complexes were precipitated by shaking overnight at 4°C using 500 μL of anti-CRP carboxyl-coated polyethylene beads (Stagnant Water Co., Osaka, Japan). The beads were washed 5 times with dilution buffer by centrifugation at 10,000×*g* for 10 min at 4°C, and the supernatant with unbound proteins was removed. The pellets were solubilized in 100 μL of triethyl-ammonium bicarbonate (TEAB) lysis buffer (20 mmol/L TEAB, 20 mmol/L DL-dithiothreitol [DTT], 1% sodium dodecyl sulfate [SDS]) at room temperature for 10 min, heated to 95°C for 10 min, and then allowed to cool for a further 10 min at room temperature. Samples containing the affinity-purified proteins were subjected to an additional centrifugation step (10,000×*g* for 30 min at room temperature), and the supernatants were collected.

#### Sodium dodecyl sulfate–polyacrylamide gelelectrophoresis (SDS-PAGE) and liquid chromatograph-mass spectrometry/mass spectrometry (LC–MS/MS) analysis

The affinity-purified proteins were applied to 8%–14% gradient gels for SDS-PAGE. After staining with 0.5% Coomassie brilliant blue G-250, the lane with a molecular weight of approximately 12 kDa, which was only present in serum from lung cancer patients and not in healthy control serum, was excised and subjected to in-gel tryptic digestion. After in-gel digestion with trypsin, the extracted peptide mixtures were loaded onto a nanoscale LC-ESI-Q-TOF MS instrument (Q-TOF Micromass Spectrometer, Waters Co., Milford, MA, USA) for protein identification. All data generated from the gel section were used to search the international protein index (IPI) human database (v3.61) using Paragon Algorithm 21, which is integrated into the Protein Pilot search engine (v.3; AB SCIEX, Foster, CA, USA).

#### Two-dimensional electrophoresis (2-DE) and matrix-assisted laser desorption ionization time of flight/time of flight mass spectrometry (MALDI TOF/TOF MS) analysis

Affinity-purified CRP complexes were subjected to two-dimensional separation, and 2-DE runs were repeated three times. After electrophoresis, the gels were stained with blue silver, scanned in a densitometer (Molecular Imager FX, BioRad company, Hercules, CA, USA) at a resolution of 600 dpi, and analyzed using PDQuest software (version 7.1.0, BioRad). Protein spots exhibiting twofold or higher change in density (Student’s *t* test, *P* < 0.05) in a consistent direction were considered to be different and selected for further identification.

#### Peptide extraction

Protein spots of interest were in-gel digested by trypsin. Gel pieces were first discolored in 50% acrylonitrile (ACN) and 25 mmol/L ammonium bicarbonate and subsequently subjected to reduction in 10 mmol/L DTT and alkylation in 55 mmol/L iodoacetic acid. Following vacuum drying, the gel pieces were incubated with sequencing grade modified trypsin (Promega Co., Madison, WI, USA) at a final concentration of 0.01 mg/mL in 25 mmol/L ammonium bicarbonate for 16 h at 37°C. Supernatants were collected, vacuum-dried, and re-dissolved in 50% ACN and 0.1% trifluoroacetic acid (TFA) for MS analysis.

#### MALDI TOF/TOF analysis

Tryptic peptides were finally dissolved in MALDI matrix (7 mg/mL α-cyano-4-hydroxycinnamic acid in 0.1% TFA and 50% ACN), spotted onto 192-well stainless steel MALDI target plates, and analyzed using an ABI 4800 Proteomics Analyzer MALDI TOF/TOF mass spectrometer (Applied Biosystems, Carlsbad, CA, USA). The MS together with the MS/MS spectra were searched against the IPI Human database version 3.24 using GPS ExplorerTM Version 3.0 software and MASCOT database search algorithms (version 2.0). The following search criteria were used: trypsin specificity, cysteine carbamidomethylation (C), and methionine oxidation (M) as variable modifications, 1 trypsin miscleavage allowed, 100 ppm MS tolerance, and 0.25 Da MS/MS tolerance. All identified proteins had protein scores greater than 59 (*P* < 0.05) and individual ion scores greater than 21 with expected values (*P* < 0.05). All MS/MS spectra were further validated manually.

### Co-immunoprecipitation (IP) of CRP-bound serum amyloid A (SAA) complexes from serum samples and cell culture media

#### CRP-SAA complex expression in cell culture media

293FT cells were maintained in our lab and cultured in dulbecco’s modified eagle medium (DMEM) (Gibco, Grand Island, NY, USA) supplemented with 10% fetal bovine serum (Gibco) and 1% penicillin/streptomycin (Gibco). For transfections, we used the pSPHis plasmid vector, pSPHis-CRP, and pSPHis-SAA recombinant plasmid which were all constructed and preserved by our laboratory, and these plasmids can effectively express secreted proteins from inserted gene fragments. A CRP-secreting 293FT stable cell line (293FT-CRP) was established through transfection of the pSPHis-CRP plasmid, and a SAA-secreting 293FT stable cell line (293FT-SAA) was established by transfection with the pSPHis-SAA plasmid. The 293FT-CRP and 293FT-SAA cell lines were mixed in equivalent numbers for culture. After incubation for 24 h, cells were washed twice with serum-free DMEM, then further incubated for 48 h in serum-free DMEM. The cell culture media were collected and centrifuged at 2,000×*g* for 20 min, and the supernatants, which contained CRP-SAA complexes, were collected.

#### Co-IP of CRP-SAA complexes

Similar to the method for purification of CRP complexes, the CRP-SAA complexes from the serum samples or the cell supernatants were evaluated by IP using anti-CRP carboxyl-coated polyethylene beads (Stagnant Water Co.), followed by immunoblotting with anti-SAA polyclonal antibody (sc-20651, Santa Cruz, Dallas, TX, USA). The CRP-SAA complexes were also evaluated by IP with anti-SAA polyclonal antibody and protein A/G beads (20423, Pierce, Rockford, IL, USA), and immunoblotting with an anti-CRP antibody (ab13426, Abcam, Cambridge, UK).

#### Western blotting

Purified CRP complexes were resolved using 12% SDS-PAGE. The proteins were then transferred to polyvinylidene difluoride (PVDF) membranes and incubated with anti-SAA or anti-CRP antibodies after blocking. The membrane was washed with phosphate-buffered saline with tween (PBST) and then incubated with horseradish peroxidase-conjugated secondary antibodies (Pierce) prior to visualization of the bands using enhanced chemiluminescence western blotting substrate (32106, Pierce).

### Immunohistochemical assay

Anti-SAA polyclonal rabbit antibody was used as the primary antibody. The immunohistochemical kit (SP-9001 rabbit SP kit, 50581654) was obtained from Zhongshan Golden Bridge Co. Ltd. (Beijing, China). The anti-SAA polyclonal antibody was incubated overnight at 4°C. After washing with phosphate-buffered saline (PBS), the biotinylated secondary antibody of the kit was applied for 15 min at 37°C. Then, the sections were incubated with streptavidin–horseradish peroxidase complex and developed with 3′-diaminobenzidine tetrahydrochloride (DAB). Light Mayer’s hematoxylin (Zhongshan Golden Bridge) was applied as a counterstain.

### Enzyme-linked immunosorbent assay (ELISA)

To detect CRP-bound SAA (CRP-SAA) or total SAA, 96-well ELISA plates (Corning, Corning, NY, USA) were coated with 0.5 μg/mL anti-CRP monoclonal antibody (DY1707, R&D, Minneapolis, MN, USA) or 100 ng/well anti-SAA polyclonal antibody in 0.05 mol/L NaHCO_3_, pH 9.0, overnight at 4°C, followed by blocking with 3% bovine serum albumin in PBS for 3 h at 37°C. Serum samples were tested in duplicate; they were diluted at 1:100 with Signal Boost Solution 1 (407207, Merck Calbiochem, Darmstadt, Hesse, Germany) and incubated for 2 h at 37°C. After four washes with PBST, the plates were incubated with 1:6,000 diluted biotin-labeled anti-SAA monoclonal antibody (LS-C20534, LifeSpan BioSciences, Seattle, WA, USA) in Signal Boost Solution 2 (407207, Merck Calbiochem) for another 2 h at 37°C. The plates were then incubated with 1:5,000 HRP-labeled avidin (43-4323, Invitrogen, Carlsbad, CA, USA). After four washes with PBST, color was developed using tetramethyl benzidine substrate, and the optical density (OD) at 450 nm was measured. Every serum sample was tested three times, and the average OD value was recorded.

For the detection of serum CRP, we chose the classic quantitative method. Serum CRP was measured by particle-enhanced immunoturbidimetry (Wako, Osaka, Japan) using a Hitachi 2008 system (Hitachi, Tokyo, Japan).

### Statistical analysis

Statistical analyses were mainly conducted using the SPSS 16.0 statistical software package (SPSS Inc., Chicago, IL, USA). Receiver operating characteristic (ROC) curve analyses were used to compare the sensitivity and specificity for the detection of CRP-SAA and total SAA. The Mann–Whitney *U* test was used to analyze associations between the levels of CRP-SAA, total SAA and clinical characteristics. Survival curves were plotted using the Kaplan–Meier method and compared using the log-rank test. The significance of various variables in survival outcomes was assessed by applying the Cox proportional hazards model to the univariate and multivariate analyses. *P* < 0.05 was considered significant in all cases.

## Results

### Identification of CRP-SAA complexes in the serum of lung cancer patients

**Fig. 1:**
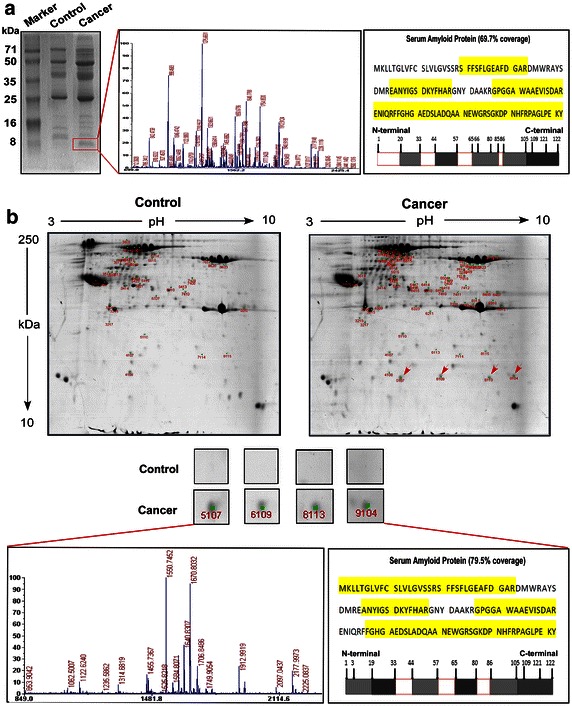
Identification of serum C-reactive protein-bound serum amyloid A (CRP-SAA) complexes in serum samples from lung cancer patients. **a** Sodium dodecyl sulfate–polyacrylamide gelelectrophoresis (SDS-PAGE) and mass spectrometry (MS) of serum samples from healthy controls and lung cancer patients. The *red frame* indicates the discrepant bands. **b** Analysis of discriminative masses between normal and cancer serum samples through two-dimensional electrophoresis (2-DE) and MS. The *red arrows* indicate the discrepant masses corresponding to SAA1/SAA2 proteins.

CRP-bound components in serum samples obtained from healthy controls or lung cancer patients were purified using anti-CRP carboxyl-coated polyethylene beads and subjected to SDS-PAGE. Gel bands show a mass of 12–14 kDa, indicating CRP complexes, in the serum samples from lung cancer patients but not in healthy control samples (Fig. 1a). These bands were selected for protein identification. The SAA peptide fragments (SAA1/SAA2) were frequently identified in subsequent LS-MS/MS analysis and covered 69.7% of the amino acid sequences of SAA (IPI00552578). These data suggested that CRP formed a complex with SAA in the serum samples from lung cancer patients.

For further confirmation, serum CRP-bound components from healthy controls or lung cancer patients were evaluated using 2-DE. The protein spots with discrepancies between healthy controls and lung cancer patients were analyzed using MALDI TOF/TOF MS (Fig. 1b). CRP-bound components with significant differential expression (twofold or more change, *P* < 0.05, Student’s *t*-test) in the serum samples from lung cancer patients are shown in Table 2. SAA1/SAA2 (IPI00552578) and SAA2 isoform A (IPI00006146) exhibited 79.5% amino acid sequence coverage in the MS analysis (Fig. 1b). Because the protein homology between SAA1 and SAA2 is 95.9%, we hereafter used SAA to indicate both SAA1 and SAA2.

**Table 2:** Differentially expressed CRP-bound proteins in lung cancer patients identified by MALDI-TOF/TOF MS/MS

Protein name	Spot no.	Accession no.	Molecular weight (Da)	Protein PI	Protein score (CI)	Total ion score (CI)	Best ion score (CI)	Peptide count	Intensity matched	Fold change*
SAA1/SAA2	5,107	IPI00552578	13,523.5	6.28	284 (100%)	212 (100%)	96 (100%)	8	19.769	+∞
	6,109	IPI00552578	13,523.5	6.28	279 (100%)	207 (100%)	98 (100%)	8	34.058	+∞
	8,113	IPI00006146	13,518.6	9.20	338 (100%)	280 (100%)	102 (100%)	7	33.873	+∞
	9,104	IPI00006146	13,518.6	9.20	143 (100%)	83 (100%)	*62 (100%)*	7	14.093	+∞
APOE	4,108	IPI00021842	36,131.8	5.65	105 (100%)	79 (100%)	38 (97.11%)	8	8.824	−10.32
	7,114	IPI00021842	36,131.8	5.65	74 (99.67%)	35 (94.84%)	24 (31.61%)	11	7.507	+2.01
	3,414	IPI00021842	36,131.8	5.65	187 (100%)	93 (100%)	36 (93.32%)	16	19.241	−2.53
KRT1	8,115	IPI00220327	65,978.0	8.16	132 (100%)	83 (100%)	43 (97.55%)	13	8.195	+2.46
	3,217	IPI00220327	65,978.0	8.16	290 (100%)	197 (100%)	66 (99.99%)	18	22.338	+3.02
	8,623	IPI00220327	65,978.0	8.16	64 (97.22%)	20 (0%)	11 (0%)	12	5.121	+2.11
CLU^a^	2,407	IPI00291262	52,461.0	5.89	331 (100%)	298 (100%)	69 (99.99%)	9	46.414	+2.06
CLU^b^	2,409	IPI00795633	52,332.0	5.97	338 (100%)	296 (100%)	91 (100%)	10	61.136	+2.02
HBA1/HBA2	7,410	IPI00410714	15,247.9	8.72	97 (99.99%)	82 (100%)	82 (99.69%)	3	16.458	+2.23
Actin^c^	3,514	IPI00922240	38,608.2	5.19	74 (99.69%)	29 (55.07%)	29 (0%)	9	5.190	+2.34
TTR	4,107	IPI00646384	13,146.4	5.34	83 (99.96%)	65 (99.99%)	65 (99.99%)	3	15.846	+2.04
VIM	7,617	IPI00418471	53,619.1	5.06	64 (96.58%)	21 (0%)	18 (0%)	13	36.297	+2.65
IGKC	8,312	IPI00430847	25,690.7	8.18	110 (100%)	83 (100%)	59 (99.98%)	5	4.978	+2.47
ENO1	8,620	IPI00465248	47,139.3	7.01	82 (99.96%)	19 (0%)	14 (0%)	12	16.301	+2.28

*CRP* C-reactive protein, *MALDI-TOF/TOF MS/MS* matrix-assisted laser desorption ionization-time of flight/time of flight mass spectrometry/mass spectrometry, *PI* isoelectric point, *CI* confidence interval, *SAA* serum amyloid A, *APOE* apolipoprotein E, *KRT1* keratin, type II cytoskeletal 1, *CLU* clusterin, *HBA* hemoglobin, *TTR* transthyretin, *VIM* vimentin, *IGKC* Ig kappa chain C region, *ENO1* enolase 1.

* Fold-change of the protein levels in serum samples from lung cancer patients to those in serum samples from healthy controls: “+∞” indicates tending to be infinity; “+” indicates up-regulation; “−” indicates down-regulation. SAA was only presented in serum samples from lung cancer patients but not in those from healthy controls, thus the fold-change tends to be infinity.

^a^Highly similar to Clusterin.

^b^cDNA FLJ57622, highly similar to Clusterin.

^c^cDNA FLJ55253, highly similar to Actin, cytoplasmic 1.

### Confirmation of CRP-SAA complexes in serum samples or cell culture media and expression of SAA in lung cancer tissues

**Fig. 2:**
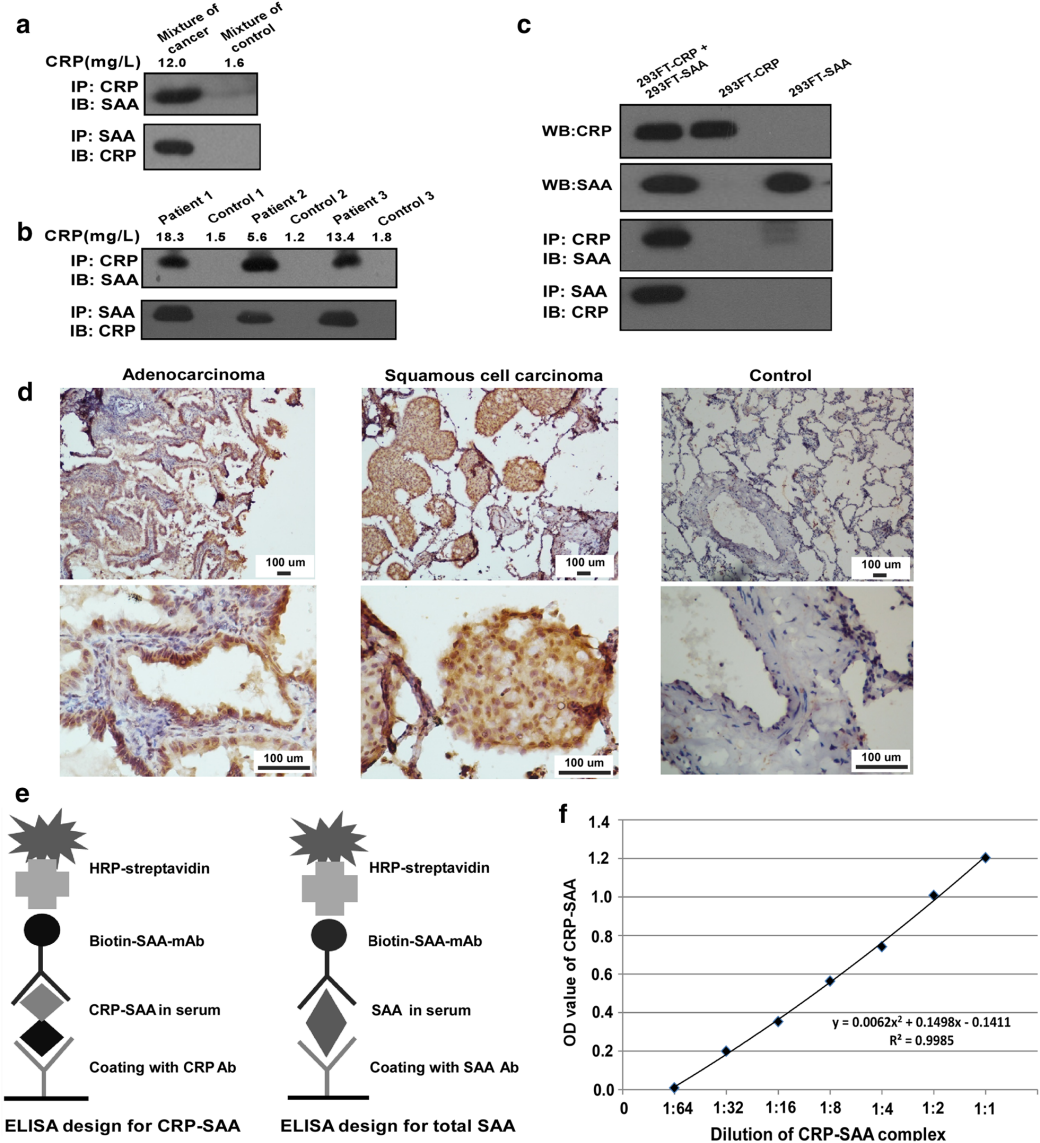
Confirmation of CRP-SAA complexes in the serum samples or cell culture media and expression of SAA in lung cancer tissues. **a** Identification of CRP-SAA complexes in mixed serum from ten healthy controls and from ten lung cancer patients through co-immunoprecipitation (co-IP). **b** Identification of CRP-SAA complexes in serum samples from individual healthy controls and lung cancer patients through co-IP. **c** Identification of CRP-SAA complexes in culture media from mixed 293FT-CRP and 293FT-SAA cells through co-IP. **d** Immunohistochemical assay shows high expression of SAA in both lung adenocarcinoma and squamous cell carcinoma tissues and low expression in normal lung tissues. **e** Enzyme-linked immunosorbent assay (ELISA) for the detection of CRP-SAA and total SAA. **f** The standard curve of the ELISA system for CRP-SAA using serially diluted culture media from mixed 293FT-CRP and 293FT-SAA cells.

SAA is known as an acute-phase protein and has been reported to be elevated in the serum of lung cancer patients [17]. We detected the existence of CRP-SAA in the mixed serum sample from 10 lung cancer patients on which we previously performed the proteomics analysis (Fig. 2a) and some serum samples from individual lung cancer patients (Fig. 2b) by co-IP. The property of CRP binding to SAA was also confirmed in the media from mixed cultivation of the 293FT-CRP and 293FT-SAA cell lines (Fig. 2c).

Recently, it was reported that melanoma cells can produce SAA, although SAA is typically known as a primary product of hepatocytes [18]. We found that SAA was highly expressed in lung cancer tissue but expressed at low levels in normal lung tissues (Fig. 2d).

Next, we investigated whether CRP-SAA or total SAA in serum could be used as a lung cancer marker. We established two ELISA systems: one to detect total SAA, in which two different SAA antibodies were used in coating and detection, and another to detect CRP-SAA, in which anti-CRP antibodies were used to capture CRP complexes and biotin-labeled anti-SAA antibodies were used to detect CRP-SAA (Fig. 2e).

To evaluate the efficacy of the ELISA for CRP-SAA detection, mixed culture media of 293FT-CRP and 293FT-SAA cells were diluted serially to prepare a standard curve for ELISA of CRP-SAA. The R value of the standard curve was 0.998,5, indicating that this ELISA system can effectively determine the levels of CRP-SAA (Fig. 2f).

### Association of elevated serum CRP-SAA level with clinical features of lung cancer

**Fig. 3:**
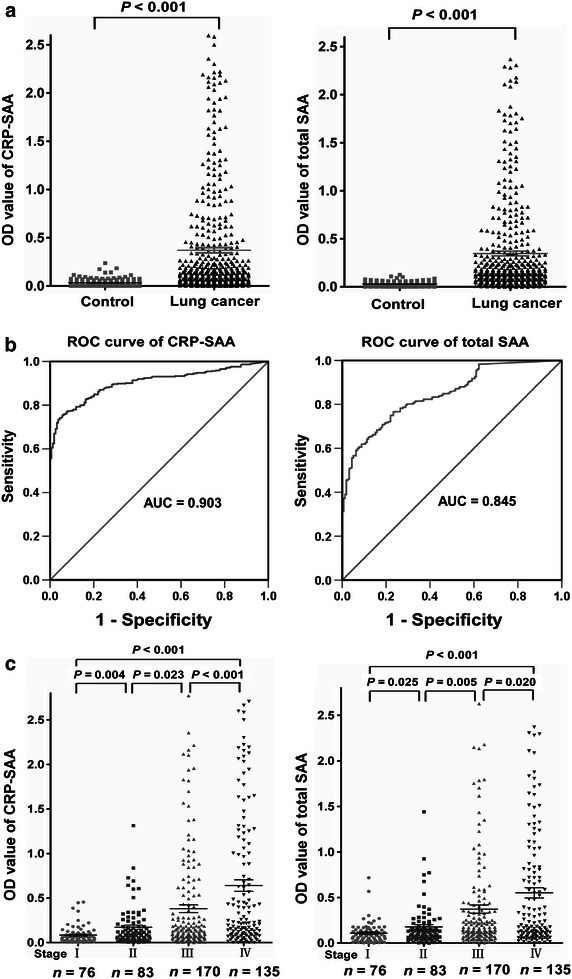
CRP-SAA is a potential diagnostic marker for lung cancer. **a** The serum levels of both CRP-SAA and total SAA in lung cancer patients (*n* = 464) are elevated significantly compared with those in healthy controls (*n* = 159). **b** Receiver operating characteristic (ROC) curve analysis shows that the area under the ROC curve (AUC) is 0.903 for CRP-SAA and 0.845 for total SAA in the diagnosis of lung cancer. **c** Patients with advanced-stage cancers had higher levels of both CRP-SAA and total SAA compared with those with early-stage cancers.

The serum levels of CRP-SAA and total SAA were evaluated in samples from two independent cohorts of lung cancer patients and healthy control samples using the two ELISA systems. Similar to total SAA, the serum levels of CRP-SAA in patients with lung cancer were elevated significantly compared with those in healthy controls (*P* < 0.001) (Fig. 3a). ROC analysis showed that the area under the ROC curve (AUC) for CRP-SAA in the diagnosis of lung cancer was 0.903, which was higher than the AUC of 0.845 obtained for total SAA (Fig. 3b). In addition, patients with advanced-stage cancers had higher levels of CRP-SAA and total SAA compared with those with early-stage cancers (Fig. 3c).

ROC analysis showed that ODs of 0.10 for CRP-SAA and 0.17 for total SAA were the optimal cutoff values to divide patients into low-level and high-level groups, respectively. Statistical analysis of clinical data between the low- and high-level groups of lung cancer patients revealed that the serum levels of CRP-SAA were closely associated with sex, smoking status, tumor size, lymph node involvement, distant metastasis, and clinical stage (Table 3). Similar to total SAA, high levels of CRP-SAA indicated severe clinical features.

**Table 3:** Association between serum levels of CRP-bound SAA (CRP-SAA), total SAA and clinical characteristics of the 464 lung cancer patients

Characteristic	CRP-SAA [cases (%)]		*P* value	Total SAA [cases (%)]		*P* value
	Low level (OD ≤ 0.10)	High level (OD > 0.10)		Low level (OD ≤ 0.17)	High level (OD > 0.17)	
Total	212	252		271	193	
Age			*0.725*			0.741
<60 years	112 (24.1)	129 (27.8)		139 (30.0)	102 (22.0)	
≥60 years	100 (21.6)	123 (26.5)		132 (28.4)	91 (19.6)	
Sex			*0.001*			0.106
Male	142 (30.6)	203 (43.8)		194 (41.8)	151 (32.5)	
Female	70 (15.1)	49 (10.6)		77 (16.6)	42 (9.1)	
Smoking status			*<0.001*			*<0.001*
No	113 (24.4)	85 (18.3)		139 (30.0)	59 (12.7)	
Yes	99 (21.3)	167 (36.0)		132 (28.4)	134 (28.9)	
Treatment			*<0.001*			*<0.001*
Multimodality therapy without operation	86 (18.5)	54 (11.6)		112 (24.1)	28 (6.0)	
Operation alone	62 (13.4)	76 (16.4)		82 (17.7)	56 (12.1)	
Operation and multimodality therapy	64 (13.8)	122 (26.3)		77 (16.6)	109 (23.5)	
T stage			*<0.001*			*<0.001*
T1	41 (8.8)	29 (6.3)		54 (11.6)	16 (3.4)	
T2	109 (23.5)	100 (21.6)		130 (28.0)	79 (17.0)	
T3	32 (6.9)	82 (17.7)		51 (11.0)	63 (13.6)	
T4	30 (6.5)	41 (8.8)		36 (7.8)	35 (7.5)	
N stage			*<0.001*			*<0.001*
N0	93 (20.0)	72 (15.5)		113 (24.4)	52 (11.2)	
N1	33 (7.1)	41 (8.8)		47 (10.1)	27 (5.8)	
N2	68 (14.7)	90 (19.4)		84 (18.1)	74 (15.9)	
N3	18 (3.9)	49 (10.6)		27 (5.8)	40 (8.6)	
M stage			*<0.001*			*<0.001*
M0	174 (37.5)	155 (33.4)		213 (45.9)	116 (25.0)	
M1	38 (8.2)	97 (20.9)		58 (12.5)	77 (16.6)	
Clinical stage			*<0.001*			*<0.001*
I	57 (12.3)	19 (4.1)		61 (13.1)	15 (3.2)	
II	41 (8.8)	42 (9.1)		60 (12.9)	23 (5.0)	
III	76 (16.4)	94 (20.3)		92 (19.8)	78 (16.8)	
IV	38 (8.2)	97 (20.9)		58 (12.5)	77 (16.6)	
Pathologic type			0.192			0.784
Adenocarcinoma	112 (24.1)	131 (28.2)		147(31.7)	96(20.7)	
Squamous cell carcinoma	65 (14.0)	65 (14.0)		72 (15.5)	58 (12.5)	
Small cell carcinoma	15 (3.2)	33 (7.1)		26 (5.6)	22 (4.7)	
Others	20 (4.3)	23 (5.0)		26 (5.6)	17 (3.7)	
Primary tumor site			0.821			0.301
Left lung	86 (18.5)	107 (23.1)		105 (22.6)	88 (19.0)	
Right lung	126 (27.2)	145 (31.3)		166 (35.8)	105 (22.6)	

The Mann-Whitney *U* test was used to analyze the associations. Tumor size, lymph node involvement, distant metastasis, and clinical stage were classified according to the seventh edition of the Union for International Cancer Control (UICC) Staging system for Lung Cancer.

*P* < 0.05 is considered significant and the *P*-value is in italics.

*OD* optical density.

### Prognostic value of CRP-SAA in the retrospective cohort of lung cancer patients

**Fig. 4:**
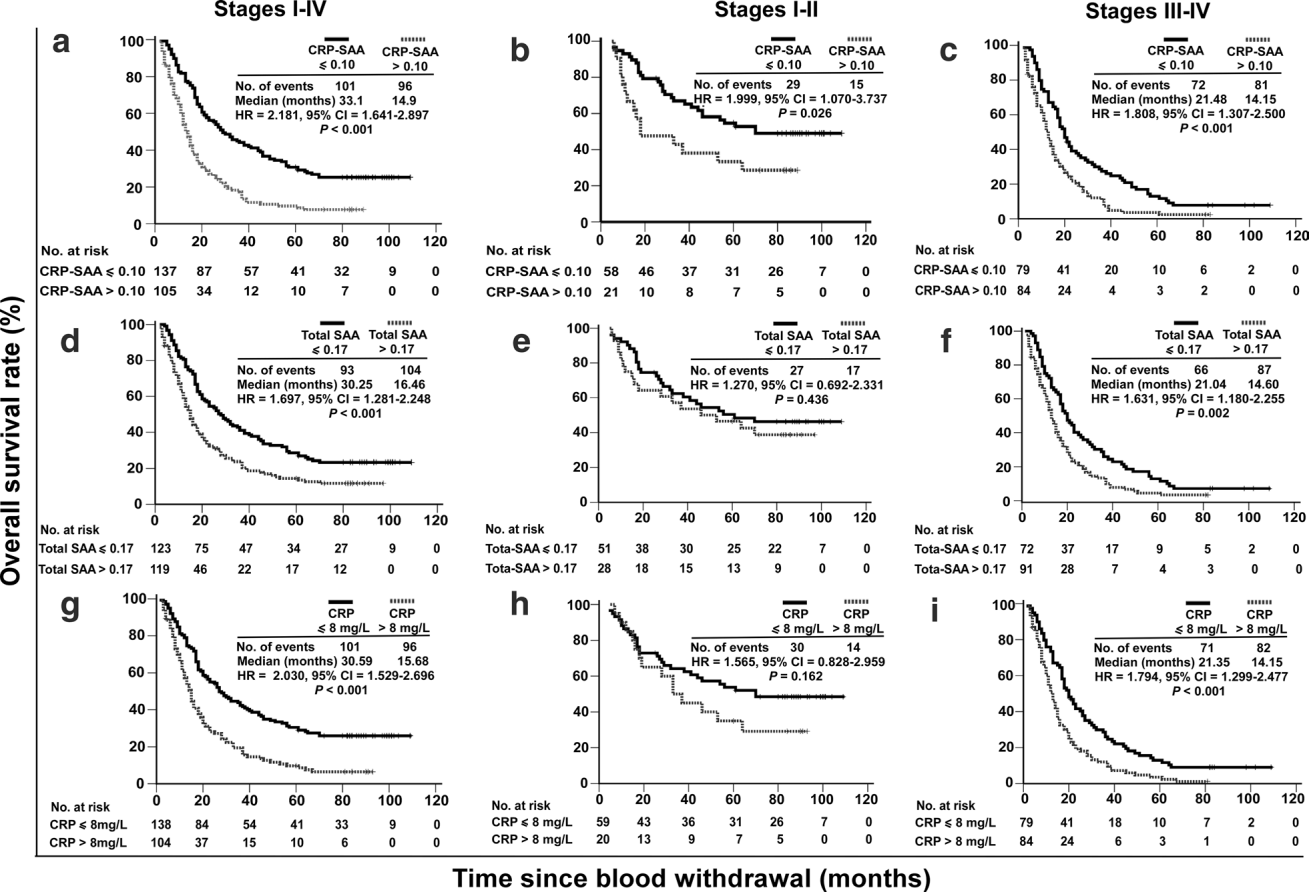
Prognostic values of serum CRP-SAA, total SAA, and CRP in the retrospective cohort of lung cancer patients. **a** Kaplan–Meier survival curves of the 242 patients divided by the cutoff optical density (OD) value for CRP-SAA into low-level (OD ≤ 0.10) and high-level (OD > 0.10) groups. **b** Survival curves of 79 patients at stages I–II divided by the cutoff value for CRP-SAA into low- and high-level groups. **c** Survival curves of 163 patients at stages III-IV divided by the cutoff value for CRP-SAA into low- and high-level groups. **d** Survival curves of the 242 patients divided by the cutoff OD value for total SAA into low-level (OD ≤ 0.17) and high-level (OD > 0.17) groups. **e** Survival curves of 79 patients at stages I–II divided by the cutoff value for total SAA into low- and high-level groups. **f** Survival curves of 163 patients at stages III–IV divided by the cutoff value for total SAA into low- and high-level groups. **g** Survival curves of the 242 patients divided by the cutoff value for CRP into low-level (CRP ≤ 8 mg/L) and high-level (CRP > 8 mg/L) groups. **h** Survival curves of 79 patients at stages I–II divided by the cutoff value for CRP into low- and high-level groups. **i** Survival curves of 163 patients at stages III–IV divided by the cutoff value for CRP into low- and high-level groups. Significant differences were calculated using a log-rank test. The numbers of patients at risk at each specific time point are indicated. The number of events indicates the cumulative number of all events during the entire follow-up period. *HR* hazard ratio calculated by univariate Cox regression analysis, not adjusted by other factors. *CI* confidence interval.

We evaluated whether CRP-SAA could be a prognostic marker for lung cancer. In the retrospective cohort, patients with high levels of CRP-SAA showed a shorter median survival than those with low levels of CRP-SAA (Fig. 4a). The 5-year overall survival (OS) rate was lower in the high-level group than in the low-level group (9.5% vs. 29.9%). When patients were stratified by cancer stages, a high level of CRP-SAA was also associated with a shorter median survival in the stages I–II (Fig. 4b) and stages III–IV subgroups (Fig. 4c; Table 4).

**Table 4:** Associations of CRP-SAA, total SAA, and CRP with survival of lung cancer patients in both the retrospective cohort and the prospective cohort

Variate	The retrospective cohort			The prospective cohort		
	Stages I–IV	Stages I–II	Stages III–IV	Stages I–IV	Stages I–II	Stages III–IV
CRP-SAA						
High level	33.1	–	21.48	–	–	–
Low level	14.9	–	14.15	–	–	–
HR	2.181	1.999	1.808	2.744	2.613	2.209
95% CI	1.641–2.897	1.070–3.737	1.307–2.500	1.810–4.161	1.211–5.635	1.336–3.652
*P* value	<0.001	0.026	<0.001	<0.001	0.01	0.001
Total SAA						
High level	30.25	–	21.04	–	–	–
Low level	16.46	–	14.6	–	–	–
HR	1.697	1.27	1.631	1.775	0.44	1.602
95% CI	1.281–2.248	0.692–2.331	1.180–2.255	1.243–2.533	0.105–1.851	1.079–2.378
*P* value	<0.001	0.436	0.002	0.001	0.246	0.016
CRP						
High level	30.59	–	21.35	–	–	–
Low level	15.68	–	14.15	–	–	–
HR	2.03	1.565	1.794	2.185	1.64	1.874
95% CI	1.529–2.696	0.828–2.959	1.299–2.477	1.534–3.112	0.774–3.474	1.238–2.836
*P* value	<0.001	0.162	<0.001	0.001	0.189	0.002

Abbreviations as in previous Tables. “–” indicates that the cumulative survival rate is below 50%. For grouping, the cutoff OD value for CRP-SAA is 0.10; the cutoff OD value for total SAA is 0.17; and the cutoff value of CRP is 8 mg/L.

Furthermore, the prognostic value of CRP-SAA was compared with those of total SAA and CRP. The cutoff value of CRP used for grouping was 8 mg/L, as determined by ROC analysis. High levels of both total SAA and CRP were associated with shorter survival in the whole cohort and the stages III-IV subgroup, but not in the stages I-II subgroup; the 5-year OS rate was lower in the high-level group than in the low-level group for both total SAA (14.3% vs. 27.6%) and CRP (9.6% vs. 29.7%) (Fig. 4d–i; Table 4).

### Prognostic value of CRP-SAA in an independent prospective cohort of lung cancer patients

**Fig. 5:**
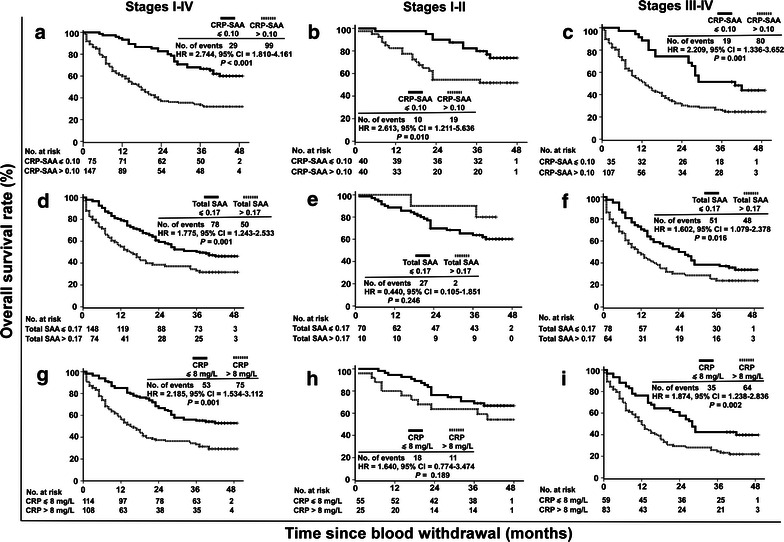
Prognostic values of serum CRP-SAA, total SAA, and CRP in the prospective cohort of lung cancer patients. **a** Kaplan–Meier survival curves of the 222 patients divided by the cutoff OD value for CRP-SAA into low- and high-level groups. **b** Survival curves of 80 patients at stages I–II divided by the cutoff value for CRP-SAA into low- and high-level groups. **c** Survival curves of 142 patients at stages III–IV divided by the cutoff value for CRP-SAA into low- and high-level groups. **d** Survival curves of the 222 patients divided by the cutoff OD value for total SAA into low- and high-level groups. **e** Survival curves of 80 patients at stages I–II divided by the cutoff value for total SAA into low- and high-level groups. **f** Survival curves of 142 patients at stages III–IV divided by the cutoff value for total SAA into low- and high-level groups. **g** Survival curves of the 222 patients divided by the cutoff value for CRP into low- and high-level groups. **h** Survival curves of 80 patients at stages I–II divided by the cutoff value for CRP into low- and high-level groups. **i** Survival curves of 142 patients at stages III–IV divided by the cutoff value for CRP into low- and high-level groups.

To confirm the prognostic value of CRP-SAA, we recruited an independent prospective cohort of patients and followed their survival for 4 years. Consistent with the results obtained from the retrospective cohort, high levels of CRP-SAA were associated with shorter survival in the whole prospective cohort, the stages I–II subgroup, and the stages III–IV subgroup; high levels of total SAA and CRP were associated with shorter survival in the whole prospective cohort and the stages III–IV subgroup, but not in the stages I-II subgroup (Fig. 5; Table 4).

### Potential prognostic value of CRP-SAA for lung cancer

The results of univariate and multivariate Cox regression analyses of the prognostic values of various factors in the 464 lung cancer patients are presented in Tables 5 and 6. Multivariable analysis, adjusted for age, sex, smoking status, clinical stage, and treatment regimen, showed that CRP-SAA was an independent prognostic factor for OS in the whole cohort, the stages I–II subgroup, and the stages III-IV subgroup (all *P* < 0.005); total SAA and CRP were also independent prognostic factors for OS in the whole cohort and the stages III–IV subgroup (all *P* < 0.005), but not in the stages I–II subgroup (all *P* > 0.05; Table 6).

**Table 5:** Univariate and Multivariate Cox regression analysis of potential prognostic factors for 464 lung cancer patients

Variable	Univariate model			Multivariate model		
	HR	95% CI	*P*	HR	95% CI	***P***
Age				–	–	–
<60 years	1.000					
≥60 years	1.151	0.926–1.432	0.206			
Sex				–	–	–
Female	1.000					
Male	1.285	0.997–1.657	0.053			
Smoking status						
No	1.000			1.000		
Yes	1.533	1.224–1.919	<0.001	1.462	1.080–1.980	0.014
Treatment						
Multimodality therapy without operation	1.000			1.000		
Operation alone	0.231	0.173–0.309	<0.001	0.568	0.367–0.880	0.011
Operation and multimodality therapy	0.326	0.250–0.425	<0.001	0.492	0.362–0.669	<0.001
T stage						
T1	1.000			1.000		
T2	1.176	0.820–1.686	0.378	1.081	0.753–1.552	0.671
T3	2.019	1.382–2.951	<0.001	1.352	0.920–1.987	0.124
T4	2.162	1.436–3.253	<0.001	1.013	0.668–1.536	0.951
N stage						
N0	1.000			1.000		
N1	1.043	0.730–1.489	0.817	0.789	0.549–1.133	0.199
N2	1.756	1.346–2.293	<0.001	0.797	0.599–1.058	0.117
N3	2.725	1.958–3.793	<0.001	1.155	0.816–1.636	0.416
M stage						
M0	1.000			1.000		
M1	3.091	2.448–3.902	<0.001	0.985	0.681–1.424	0.936
Clinical stage						
I	1.000			1.000		
II	3.742	2.213–6.325	<0.001	3.254	1.909–5.546	<0.001
III	5.267	3.239–8.563	<0.001	3.435	2.013–5.864	<0.001
IV	11.25	6.855–18.461	<0.001	5.685	3.063–10.549	<0.001
CRP-SAA						
Low level	1.000			1.000		
High level	2.097	1.673–2.628	<0.001	1.545	1.223–1.951	<0.001
Total SAA						
Low level	1.000			1.000		
High level	1.778	1.429–2.212	<0.001	1.329	1.061–1.663	0.013
CRP						
Low level	1.000			1.000		
High level	2.045	1.641–2.549	<0.001	1.454	1.156–1.829	0.001

“−”, age and sex are not included in the Multivariate Cox regression analysis. Other footnotes as in Table 4.

**Table 6:** Stratified Cox regression analysis of CRP-SAA, Total SAA, and CRP for 464 lung cancer patients

Variable	Univariate model			Multivariate model^a^		
	HR	95% CI	*P*	HR	95% CI	*P*
Stages I–II						
CRP-SAA						
Low level	1.000			1.000		
High level	2.583	1.873–3.560	*<0.001*	1.677	1.188–2.368	*0.003*
Total SAA						
Low level	1.000			1.000		
High level	1.581	1.164–2.150	*0.003*	1.180	0.859–1.620	0.307
CRP						
Low level	1.000			1.000		
High level	2.113	1.563–2.857	*<0.001*	1.429	1.036–1.971	0.063
Stages III–IV						
CRP-SAA						
Low level	1.000			1.000		
High level	1.899	1.483–2.431	*<0.001*	1.594	1.237–2.052	*<0.001*
Total SAA						
Low level	1.000			1.000		
High level	1.879	1.485–2.377	*<0.001*	1.359	1.065–1.735	*0.014*
CRP						
Low level	1.000			1.000		
High level	1.970	1.554–2.499	*<0.001*	1.507	1.178–1.929	*0.001*

^a^Each serum marker was analyzed separately in the model, adjusting for age, sex, smoking status, clinical stage, and treatment. Abbreviations as in previous tables.

## Discussion

Through a differential proteomic analysis, we found that several proteins were significantly up-regulated in the form of CRP-bound complexes in the serum of lung cancer patients. SAA was exclusively presented in the form of CRP-bound complexes in the serum of lung cancer patients. Further in vitro studies confirmed that CRP could bind to SAA. SAA is known to be an acute-phase protein and is more sensitive than CRP for predicting the prognosis of cancer. Recently, many reports have identified close relationships between elevated SAA in serum and worse prognoses of several types of cancers [19–22]. SAA was reported to be a potential diagnostic and prognostic biomarker for lung cancer [23, 24]. In our study, we first found that CRP bound to SAA and formed complexes in the serum of lung cancer patients. A sandwich ELISA was developed to detect serum CRP-SAA levels and evaluate their prognostic value.

Similar to total SAA, high levels of CRP-SAA were closely associated with the clinical features of lung cancer patients. Moreover, similar to total SAA and CRP, high levels of CRP-SAA were associated with shorter survival in both retrospective and prospective cohorts of lung cancer patients. Our data agree with the results of previous studies in which increased SAA and CRP levels were found to be useful biomarkers for the prediction of lung cancer prognosis [17, 24]. Remarkably, in stages I–II patients, only CRP-SAA and not total SAA or CRP significantly predicted OS. Moreover, univariate and multivariate Cox analyses also showed that only CRP-SAA could be used as an independent prognostic marker for early-stage patients.

The performance of CRP-SAA in lung cancer prognosis may be related to its biological functions. The roles of SAA in the context of tumorigenesis include binding to extracellular matrix (ECM) components [25], enhancing plasminogen activation [26], and stimulating matrix metalloproteinase (MMP) production [27]. A recent study reported that the overexpression of SAA could promote Lewis lung carcinoma cell metastasis and lung colonization in animal models and that the expression of SAA was induced when lung cancer cells were co-cultured with macrophages or cytokines [28]. De Santo et al. [18] reported that melanoma cells produce SAA, which facilitates tumor growth by inducing neutrophils to secrete interleukin-10 (IL-10), resulting in a suppressive immune response. Because CRP can recognize both Fc α receptor I (FcαRI) and Fc γ receptor (FcγR) on macrophages, we presume that the CRP-SAA complex could help SAA to bind to macrophages and induce suppressive immune responses or promote MMP production. These properties may increase the importance of SAA or CRP-SAA in tumor pathogenesis and metastasis. In our study, we also found that SAA was highly expressed in lung cancer tissues; therefore, we presume that circulating CRP may easily contact SAA produced by lung cancer cells to form the CRP-SAA complex, and the appearance of CRP-SAA in the serum could be a predictive biomarker for lung cancer progression. Moreover, high serum levels of CRP-SAA but not total SAA or CRP in the present study were significantly associated with a worse prognosis in patients with early-stage lung cancer, suggesting that the elevation of serum CRP-SAA levels may result from both the increased production of SAA in the tumor microenvironment and the elevated production of CRP by the liver in response to the chronic inflammation of the tumor site in early stages. Therefore, CRP-SAA levels could be considered a more sensitive and relevant indicator of early lung cancer progression than CRP or SAA alone.

In addition to lung cancer, elevated levels of CRP and SAA have been observed in other types of cancer, including gastric cancer [29], esophageal squamous cell carcinoma [18], and breast cancer [30]. It would thus be interesting to determine whether CRP-SAA could serve as a potential prognostic marker in these cancers. Moreover, for potential clinical application, it is important to determine whether the elevated CRP-SAA level is restricted to cancer patients or whether it is associated with other chronic inflammatory diseases.

In conclusion, the present study reveals that serum CRP-SAA isolated from serum CRP-bound complexes is a potential marker for poor prognosis in lung cancer patients. The prognostic value of CRP-SAA is higher than that of CRP and SAA individually, especially for early-stage lung cancer patients.
